# Synthesis, characterisation, and feasibility studies on the use of vanadium tellurate(vi) as a cathode material for aqueous rechargeable Zn-ion batteries†

**DOI:** 10.1039/d2ra01166b

**Published:** 2022-04-22

**Authors:** Mangayarkarasi Nagarathinam, Cindy Soares, Yue Chen, Valerie R. Seymour, Vlastimil Mazanek, Mark A. Isaacs, Zdenek Sofer, Oleg Kolosov, John M. Griffin, Nuria Tapia-Ruiz

**Affiliations:** Department of Chemistry, Lancaster University LA1 4YB UK n.tapiaruiz@lancaster.ac.uk; Department of Physics, Lancaster University LA1 4YB UK; Department of Inorganic Chemistry, University of Chemistry and Technology Prague Technicka 5 166 28 Prague 6 Czech Republic; EPSRC National Facility for XPS (HarwellXPS) Research Complex at Harwell Didcot OX11 0FA UK

## Abstract

Aqueous rechargeable zinc-ion batteries (AZIBs) have drawn enormous attention in stationary applications due to their high safety and low cost. However, the search for new positive electrode materials with satisfactory electrochemical performance for practical applications remains a challenge. In this work, we report a comprehensive study on the use of the vanadium tellurate (NH_4_)_4_{(VO_2_)_2_[Te_2_O_8_(OH)_2_]}·2H_2_O, which is tested for the first time as a cathode material in AZIBs.

## Abstract 2

(NH_4_)_4_{(VO_2_)_2_[Te_2_O_8_(OH)_2_]}·2H_2_O is tested as a cathode in an aqueous Zn-ion battery for the first time, showing a discharge capacity of 283 mA h g^−1^ in half-cells and excellent capacity retention (91%) in concentration cells after 20 cycles.

## Graphical abstract

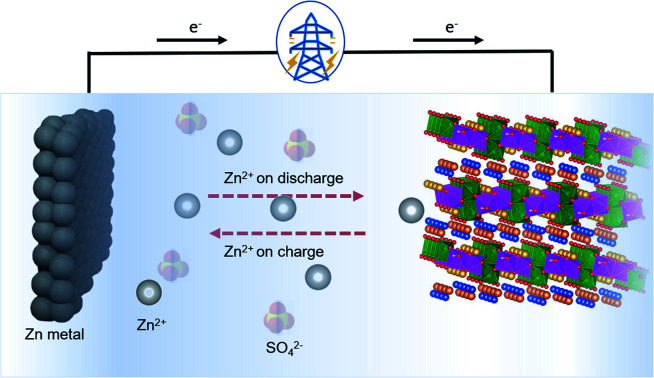

## 1. Introduction

**Fig. 1:**
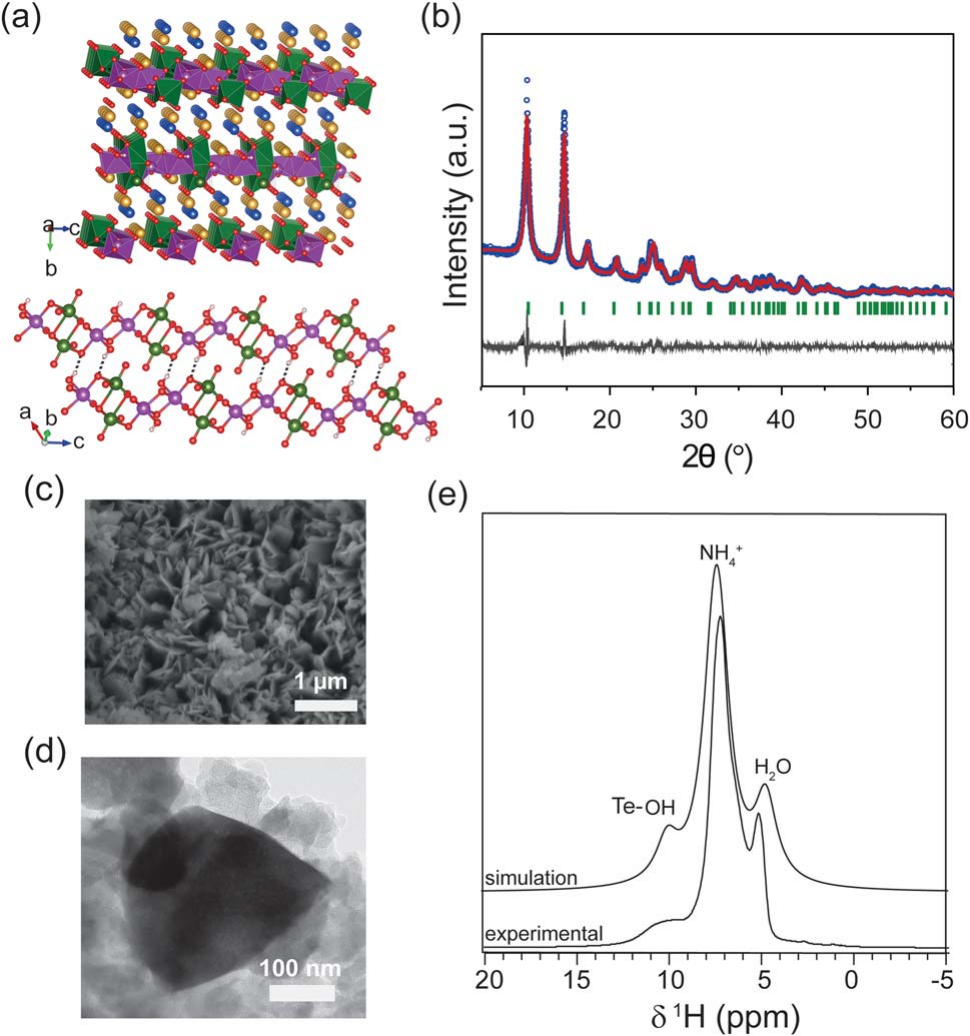
(a) Crystal structure of layered VTe, where H atoms of H_2_O (blue) and NH_4_^+^ (yellow) molecules are omitted for clarity (top figure); and ({[VO_2_]_2_[Te_2_O_8_(OH)_2_]}^4−^) anionic chains linked by Te–O–H···O–Te bonds along the *ac* plane. O–H⋯O bonds are shown as dotted lines and NH_4_^+^ and H_2_O molecules are removed for clarity (bottom figure). V atoms/VO_6_ polyhedra (green), Te atoms/TeO_6_ polyhedra (pink) and O atoms (red). (b) Experimental powder XRD data of the as-synthesized VTe product. The observed data (blue), calculated data using the Rietveld method (red), difference between experimental and calculated profiles (grey) and Bragg positions (green vertical bars) are shown. (c) FESEM image of VTe, (d) TEM image of VTe, and (e) DFT-simulated and experimental ^1^H MAS NMR spectra of VTe.

Rechargeable aqueous zinc-ion batteries (AZIBs) are a promising alternative to lead–acid batteries in grid energy storage systems.^1–4^ This is owing to the unique properties of Zn, which include a two-electron redox reaction, high theoretical gravimetric capacity (820 mA h g^−1^), low redox potential (−0.76 V *vs.* standard hydrogen electrode (SHE)), low cost and abundance.^5,6^ Furthermore, AZIBs are risk-free and easier to scale up than the classic alkali-ion batteries, due to their non-toxicity, the use of a highly stable Zn metal anode, and non-flammable and non-volatile aqueous electrolytes.^7–10^ Nevertheless, the success of this technology remains a challenge, as AZIBs require high demanding conditions, such as multivalent electrode materials with large interlayer spacing, and robust architectures to withstand the huge stress imparted upon reversible solvated Zn^2+^ ion intercalation.^7,9^ To date, significant efforts have been made to develop new manganese and vanadium-based cathode materials, where the latter have demonstrated impressively high capacities (∼300 mA h g^−1^),^9,11–15^ superior rate performance and prolonged cycle life, albeit with low operation voltages. In the pursuit of finding alternative vanadium-based electrode systems with improved electrochemical properties in AZIBs, we have investigated the use of (NH_4_)_4_(VO_2_)_2_Te_2_O_8_(OH)_2_·2H_2_O as an electrode material, here abbreviated as VTe, which was first synthesised hydrothermally at 150 °C for 3 days by Hyejin *et al.* more than a decade ago.^16^ VTe consists of 1D infinite anionic {[VO_2_]_2_[Te_2_O_8_(OH)_2_]}^4−^ chains separated by NH_4_^+^ cations and H_2_O molecules, which act as strong pillars to keep structural integrity (Fig. 1a). Electrode materials with polyanionic groups such as phosphates, silicates and sulfates have been reported to have higher electrochemical potential and improved thermal stability than their corresponding oxide counterparts.^17,18^ Thus, we will investigate the inductive and resonance effect of the [Te_2_O_8_(OH)_2_]^−6^ polyanionic moiety in the redox potential of the vanadium oxide system and explore the redox activity of the Te ions upon Zn^2+^ ion insertion/extraction. This is the first example of a vanadium tellurate(vi) compound synthesised at room temperature and tested as a cathode in AZIBs, only preceded by studies on the high-voltage K_2_Ni_2_TeO_6_ cathode used in potassium-ion batteries,^19^ and Na_2_M_2_TeO_6_ compounds (M = Ni, Co, Zn, Mg), which showed high ionic conductivities (4–11 S m^−1^) at 300 °C.^20,21^

## 2. Experimental section

### 2.1 Synthesis of VTe

V_2_O_5_ (0.270 g, 1.5 mmol) and H_6_TeO_6_ (0.690 g, 3.0 mmol) were added to 10 ml of H_2_O and stirred in a beaker until a homogeneous mixture was achieved. Then, 0.42 ml of 35% NH_4_OH solution was added dropwise with constant stirring to this solution. The yellow-orange reaction mixture was heated at 80 °C for 2–3 min until a clear solution was observed. Then, the reaction mixture was stirred at room temperature for 24 h and the solution was filtered to obtain a yellow precipitate. The yellow precipitate was washed with water and then dried at 60 °C under vacuum (0.850 g, 82% yield). All the chemicals were used as received from Sigma Aldrich without further purification. All the solvents used were of reagent grade.

### 2.2 Characterisation

Powder X-ray diffraction (PXRD) data were collected at ambient temperature using a Rigaku Smartlab X-ray diffractometer with a 9 kW Cu source generator (λ K_α1_ = 1.54051 Å) equipped with a high-resolution Vertical θ/θ 4-Circle Goniometer and a D/teX-ULTRA 250 high-speed position sensitive detector system. The crystal structure obtained by PXRD data were refined by the Rietveld refinement method using the TOPAS R2-1 software program.^22^

Fourier transform infrared (FTIR) spectra were collected in the 400–4000 cm^−1^ range with a resolution of 8 cm^−1^ using a Shimadzu IRTracer-100 FTIR spectrophotometer. Spectra were collected as the sum of 36 scans. Samples were prepared by mixing the VTe powders with KBr (Sigma Aldrich, 99%) using an agate mortar and pestle and then pressing the mixture into pellets at 5 tonnes per cm^2^.

Elemental analysis was carried out on a Vario MICRO Cube instrument in CHNS analysis mode with a program designed for measuring 2 mg samples. For the measurement, samples were weighed directly into Sn boats using a high-precision balance. Oxygen was injected into the sample for 70 s and He was used as a carrier gas. The system was zeroed by running blank samples and then it was subsequently calibrated using a sulfanilamide (C_6_H_8_N_2_O_2_S) standard. Elemental analysis data for H_22_N_4_O_16_Te_2_V_2_ (in %): H 3.31; N 8.05. (Theoretical (in %): H 3.21, N 8.11)

Thermogravimetric analyses (TGA) were carried out using a Thermys Analyzer (Setaram) with a DTA protected sensor. The sample was placed in an alumina crucible and heated from room temperature to 700 °C in an argon atmosphere at a heating rate of 10 °C min^−1^.

The microstructure of the samples was analysed using field emission scanning electron microscopy (FESEM), where the images were acquired using a JEOL JSM-7800F operating at 10.0 kV. Elemental analysis was performed at 12.0 kV using an X-ray Energy Dispersive Spectrometer (EDS) (X-Max50, large area 50 mm^2^ Silicon Drift Detector (SDD) from Oxford Instruments). Samples were placed onto carbon tabs (G3348N, Agar Scientific) attached to the metal holder and coated with a thin layer of gold using a Quorum Q150RES sputter coater (Quorum Technologies Ltd) to increase their conductivity.

Transmission electron microscopy (TEM) images and selected area electron diffraction (SAED) patterns were obtained using an EFTEM 2200 FS microscope (JEOL). A 200 keV acceleration voltage was used for TEM data collection. Sample preparation was attained by drop-casting the suspension (1 mg ml^−1^ in water) on a TEM grid (Cu; 200 mesh; formvar/carbon) and then drying it at 60 °C for 12 h.

^1^H and ^15^N solid-state NMR spectra were obtained at 16.4 T on a Bruker Avance III spectrometer operating at Larmor frequencies of 700.1 and 71.0 MHz, respectively. For ^1^H experiments, powdered samples were dried at 100 °C under vacuum for 24 h and packed into 2.5 mm rotors and spun at 30 kHz. For ^15^N experiments, powdered samples were packed into 4 mm rotors and spun at 12.5 kHz. ^1^H spectra were acquired using a DEPTH pulse sequence to remove the background signal, with a recycle interval of 10 s. ^15^N spectra were acquired using cross polarisation from ^1^H. ^125^Te solid-state NMR spectra were obtained at 9.4 T on a Bruker Avance III 400 MHz spectrometer operating at a Larmor frequency of 126.2 MHz. Powdered samples were packed into 4 mm rotors and spun at 14 kHz. ^125^Te spectra were acquired using a single 90° pulse with a repeat interval of 360 s. Spectra were referenced *via* secondary solid references of alanine (NH_3_*δ*_iso_ = 8.5 ppm), glycine (NH_3_*δ*_iso_ = −347.4 ppm), and Te(OH)_6_ (higher frequency peak *δ*_iso_ = 692.2 ppm) for ^1^H, ^15^N and ^125^Te, respectively.

^1^H chemical shifts were calculated using density functional theory (DFT). A calculation was carried out on the (NH_4_)_4_{(VO_2_)_2_[Te_2_O_8_(OH)_2_]}·2H_2_O crystal structure (ICSD 416841) using the CASTEP code. The structure was fully geometry optimised before calculation of the NMR parameters, using the GIPAW formalism^23^ as implemented within CASTEP.^24,25^ Chemical shieldings were referenced *via* a separate calculation on l-alanine (reference shielding = 30.5 ppm). Calculated ^1^H chemical shifts are shown in Table S1.† Chemical shifts for crystallographically-distinct protons within H_2_O molecules and NH_4_^+^ ions are averaged under the assumption of fast dynamic exchange between these positions.

### 2.3 Electrochemical tests

To fabricate the working electrodes, active material, super P carbon black and binder (Kynar 2801) in the weight ratio of 70 : 20 : 10 were thoroughly mixed with an agate mortar and pestle. The powdered mixture was then added to a vial and a few ml of *N*-methyl-2-pyrrolidone (NMP) were added to the mixture to make an electrode slurry. The slurry was stirred for 8 h at room temperature. 1.5 cm diameter electrodes with a thickness of 20 μm were prepared by casting the slurry onto Ti foil (used as a current collector) with a doctor-blade, and punching the electrode-coated foil with a heavy-duty manual puncher. The mass loading of the active material was typically 2–3 mg cm^−2^.

The electrochemical properties of VTe were evaluated using CR2032 stainless steel coin cells. Zn metal (180 μm thickness) was used as the counter and the reference electrode, glass microfiber filter (GB-100R, Whatman) as the separator, and a 3 M ZnSO_4_·7H_2_O aqueous solution as the electrolyte. Coin cells were aged at room temperature for 6 h before data collection took place. Galvanostatic charge/discharge cycling (GC) at different current rates and voltage windows were carried out using a Neware battery cycler (current range: 1–10 mA). Cyclic voltammetry (CV) data were collected at room temperature using an Iviumstat instrument (Ivium, Alvatek). Data were collected using a scan rate of 0.2 mV s^−1^ in the voltage window of 0.4–1.4 V *vs.* Zn^2+^/Zn.

For the preparation of the concentration cells, VTe half-cells were discharged to 0.2 V to insert Zn^2+^ ions in the VTe electrode. The electrodes were removed in an open atmosphere from the discharged cells, paired immediately with a fresh VTe electrode and assembled in a CR2032 coin cell using a glass microfiber filter as the separator and an aqueous 3 M ZnSO_4_ solution as the electrolyte. The mass of the electrodes of the two cells was kept similar and the specific capacity is calculated using the pristine VTe electrode. Galvanostatic charge/discharge cycling of the concentration cells at a constant current of 10 mA g^−1^ in the voltage range of −1.7–1.5 V was carried out at room temperature using an Iviumstat instrument (Ivium, Alvatek). The concentration cell has an open-circuit voltage of −0.411 V and it is initially charged to 1.5 V and then discharged to −1.7 V continuously.

### 2.4 *Ex situ* X-ray photoelectron spectroscopy

X-ray photoelectron spectroscopy (XPS) data on ex situ samples were acquired using a Kratos Axis SUPRA fitted with monochromated Al kα (1486.69 eV) X-rays operating at 15 mA emission and 12 kV HT (180 W) and a spot size/analysis area of 700 × 300 μm. The instrument was calibrated to gold metal Au 4f (83.95 eV) and dispersion adjusted to give a binding energy of 932.6 eV for the Cu 2p_3/2_ line of metallic copper. Ag 3d_5/2_ line FWHM at 10 eV pass energy was 0.544 eV. Source resolution for monochromatic Al Kα X-rays was ∼0.3 eV. The instrumental resolution was determined to be 0.29 eV at 10 eV pass energy, using the Fermi edge of the valence band for metallic silver. Resolution with the charge compensation system was determined to be <1.33 eV FWHM on PTFE. High-resolution spectra were obtained using a pass energy of 20 eV, step size of 0.1 eV and sweep time of 60 s, resulting in a line width of 0.696 eV for Au 4f_7/2_. Survey spectra were obtained using a pass energy of 160 eV. Charge neutralisation was achieved using an electron flood gun with filament current = 0.38 A, charge balance = 2 V, and filament bias = 4.2 V. Successful neutralisation was adjudged by analysing the C 1s region, wherein a sharp peak with no lower binding energy structure was obtained. Spectra have been charge corrected to the main line of the carbon 1s spectrum (adventitious carbon) set to 284.8 eV. All data was recorded at a base pressure of below 9 × 10^−9^ Torr and room temperature of 294 K. Data were analysed using CasaXPS v2.3.19PR1.0. Peaks were fit with a Shirley background before component analysis.^26^ The samples were transferred from the glovebox using a custom vacuum sealed transfer arm.

### 2.5 Quartz crystal microbalance experiments

Quartz crystal microbalance (QCM) experiments were performed on an eQCM 10M quartz crystal microbalance (GAMRY). Electrodes were prepared by coating an Au-coated quartz crystal (QCM5140CrAu120-050-Q, 5 MHz, 14 mm sensor diameter, 12 mm front/5 mm back electrode diameter) with a mixture of the VTe powder (active material), super P carbon black (conductive agent), and Kynar 2801 (binder) in a weight ratio of 58 : 17 : 25. Then, the electrode was heated at 60 °C for 24 h. The thickness of the composite electrode was *ca.* 20 μm. Operando EQCM was conducted during cyclic voltammetry tests. The mass change of the VTe-coated QCM electrode, Δ*m*, was calculated from the change in resonance frequency (Δ*f*), using the Sauerbrey eqn (1):1
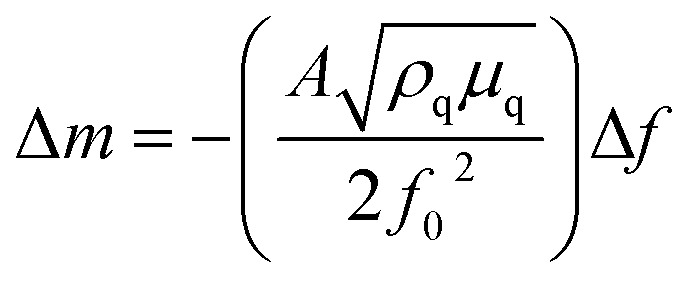
where *f*_0_ is the resonance frequency of the QCM electrode before the electrochemical tests, *A* is the ratio of the top electrode area to the QCM effective sensing area, ,*ρ*_q_ is the density of quartz (2.648 g cm^−3^), and *μ*_q_ is the shear modulus of quartz (2.947 × 10^11^ g cm^−1^ s^−2^).^27^

The number of moles of electrons that participate in the charge transfer (*N*), can be calculated by applying Faraday's law (eqn (2)):2
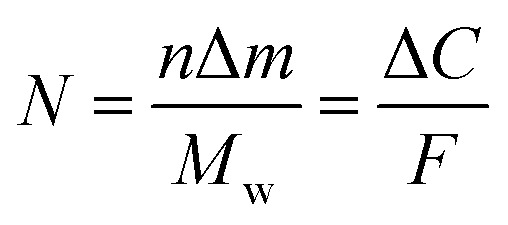
where Δ*C* is the charge passed corresponding to the Δ*m* mass change during electrolysis, *M*_w_ is the molecular weight, *F* is Faraday's constant, 96 485 C mol^−1^ and *n* is the valence number of Zn.

Eqn (2) can be then written as:3
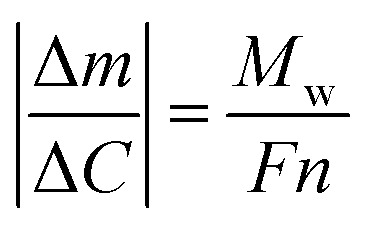


By plotting the Δ*m vs.* Δ*C*, the molecular weight of the exchanged ion can be calculated from the slope value according to the eqn (3).

### 2.6 Post-mortem studies

For post-mortem studies, electrodes were electrochemically cycled at different states of charge and then coin cells were disassembled and the electrodes washed with deionised water a few times under a steady flow of nitrogen gas. Electrodes were transferred immediately to a glovebox antechamber which was first flushed with Ar gas 5 times and then dried under vacuum at room temperature. For XPS measurements, electrodes were packed in the glovebox under Ar atmosphere. Electrodes were in contact with air during PXRD, FTIR and, FESEM data collection.

## 3. Results and discussion

(NH_4_)_4_{(VO_2_)_2_[Te_2_O_8_(OH)_2_]}·2H_2_O (VTe), was obtained using a modified synthetic procedure from the literature (see Experimental section).^16^ Rietveld analysis on the XRD data showed that the structure crystallises in a monoclinic *P*2_1_/*n* space group with *a* = 7.37(1) Å, *b* = 17.12(2) Å and *c* = 7.37(1) Å, *β* = 118.70(3)°, in agreement with the literature (Fig. 1b).^16^ The structure of VTe consists of 1D infinite anionic {[VO_2_]_2_[Te_2_O_8_(OH)_2_]}^4−^ chains along the *c* direction with edge-shared VO_6_ and TeO_5_(OH) octahedral units that form V_2_O_10_ and Te_2_O_8_(OH)_2_ dimers. These chains are connected by an extended hydrogen network of N–H⋯O and O–H⋯O bonds (originated by the presence of NH_4_^+^ cations and H_2_O molecules in the structure), and O–H⋯O hydrogen bonds from the OH group of the TeO_5_(OH) dimers (Fig. 1a). No impurities were observed in the diffraction data.

The large interlayer spacing of 8.6 Å in VTe allows a rapid migration of Zn^2+^ ions along the *ac* plane. The intralayer spacing compares well to other ammonium vanadate systems with excellent electrochemical performance due to their large ion diffusion channels, such as NH_4_V_4_O_10_ and (NH_4_)_0.5_V_2_O_5_.^28,29^ Elemental analysis confirmed the percentage of nitrogen and hydrogen as in the proposed molecular formula for VTe (see Experimental section), while EDX analysis confirmed that the V : Te and V : N elemental ratio is nearly equal to 1 : 1 and 1 : 2, respectively (Fig. S1†). Thermogravimetric analysis data showed a mass loss of 23.4% between R.T. and 410 °C, which agrees with the removal of all the H_2_O and NH_3_ molecules in the compound (Fig. S2†). SEM/TEM images in Fig. 1c and d showed that the VTe sample consists of 200–500 nm platelets. The TEM image in Fig. 1d showed that the platelets have smooth edges, and its corresponding ring-like SAED pattern (Fig. S3†) revealed the polycrystalline nature of the sample. The ^1^H MAS NMR spectrum of VTe showed three main resonances at 10, 7 and 5 ppm. Based on their relative intensities and chemical shifts, these were attributed to OH groups, pillared NH_4_^+^ ions and H_2_O molecules, respectively (Fig. 1e). DFT calculations on the published crystal structure^16^ support this assignment (Fig. 1e). The OH resonance was slightly broader than the NH_4_^+^ and H_2_O resonances owing to stronger dipolar interactions, which reduced its apparent intensity in the experimental spectrum.

The ^15^N MAS NMR spectrum for the pristine material showed a single peak at −357 ppm, which contrasts with the two crystallographic NH_4_^+^ environments in the crystal structure (Fig. S4†). DFT calculations predicted a small chemical shift difference of 6 ppm between the two N sites, which is less than the ∼8 ppm linewidth in the experimental spectrum. This explains the inability to distinguish between these two sites. The ^125^Te MAS NMR spectrum showed two distinct resonances of similar intensity at 765 and 740 ppm (Fig. S5†). This is inconsistent with the published crystal structure, which shows a single Te site. An explanation for this might be a small break in the symmetry of VTe due to the loss of H_2_O molecules.

**Fig. 2:**
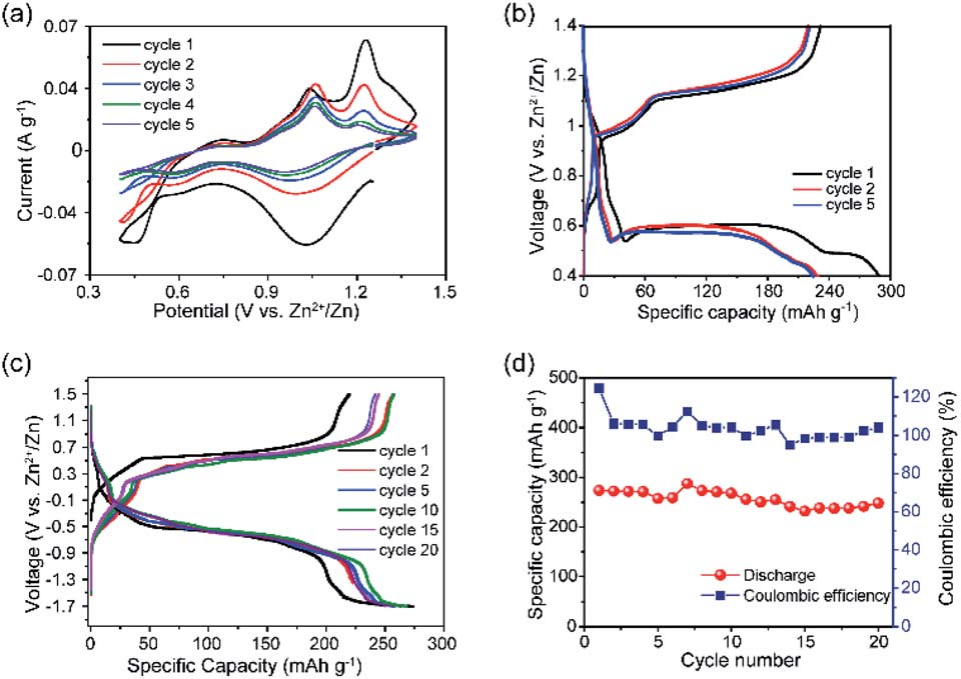
(a) CV data of VTe in the 0.4–1.4 V *vs.* Zn^2+^/Zn voltage window, using a 0.2 mV s^−1^ scan rate. (b) GC data of VTe in Zn half-cells in the 0.4–1.4 V *vs.* Zn^2+^/Zn voltage window, using a current density of 10 mA g^−1^. (c) GC data of concentration (VTe‖3 M ZnSO_4_ aq.‖Zn_*x*_VTe) cells in the −1.7–1.5 V voltage window at a current density of 10 mA g^−1^ for the 1^st^, 2^nd^, 5^th^, 10^th^, 15^th^, and 20^th^ cycles; and (d) specific capacity *vs.* cycle number plot with coulombic efficiencies for concentration (VTe‖3 M ZnSO_4_ aq.‖Zn_*x*_VTe) cells over 20 cycles.

Electrochemical studies were conducted to assess the performance of VTe as a cathode material in AZIBs. CV data showed a multistep Zn^2+^ ion insertion/extraction process, where the first scan showed cathodic peaks at 1.04 and 0.59 V and anodic peaks at 0.75, 1.06 and 1.23 V, which correspond to reversible reactions involving V^5+^/V^4+^ and V^4+^/V^3+^ redox couples (Fig. 2a). These peaks were assigned by direct comparison to other vanadate systems.^28,30^ Peaks at 1.06 and 1.23 V might be attributed to the extraction of Zn^2+^ ions from energetically different crystallographic sites during V^4+^/V^5+^ oxidation.^31^ GC data in the 0.4–1.4 V voltage window showed three discharge plateaus at *ca.* 0.90 V, 0.57 V and 0.49 V, totalling a capacity of 287 mA h g^−1^ (Fig. 2b, and magnified view in Fig. S6†). Upon charge, the capacity was not be fully retrieved (232 mA h g^−1^), due to the irreversible nature of the lowest voltage plateau, leading to an initial coulombic efficiency (CE) of *ca.* 80%. A progressive capacity decay occurred with increasing cycle number, leading to a discharge capacity of 141 mA h g^−1^ in the 20^th^ cycle (Fig. S7†). FESEM images of the VTe electroded revealed particle agglomeration after the 20^th^ cycle, which could be one of the reasons for poor cycling performance (Fig. S8†). GC tests were run using different voltage windows to optimise the long-term cycling stability of VTe without any further improvement on the cycling stability (Fig. S9†). Furthermore, rate capability studies in the 0.4–1.2 V voltage window using 10–340 mA g^−1^ current rates are shown in Fig. S10.† Although the performance of VTe was not remarkable at high rates, several approaches previously used with other Zn cathodes might be followed to improve Zn^2+^ insertion/extraction kinetics, *e.g.*, further structure tuning and mixing VTe with conducting carbon-based materials.^32,33^ This work, however, falls outside the remit of the work presented in this article.

Electrochemical tests using concentration (VTe‖3 M ZnSO_4_ aq.‖Zn_*x*_VTe) cells (Fig. 2c) were run to disregard parasitic reactions arising from the use of Zn as a counter electrode, *e.g.*, uncontrolled dendrite formation and Zn passivation, which could be the cause for the capacity decay observed in Zn half-cells.^34^ Load curves showed well-defined, long, and reversible voltage plateaus at *ca.* 0.5 and −0.5 V upon charge and discharge, respectively. The charge capacity increased from 219 to 258 mA h g^−1^ from the first to the second cycle, while the discharge capacity remained the same (271 mA h g^−1^). A reversible discharge capacity of 251 mA h g^−1^ was observed after 20 cycles, which corresponds to an excellent capacity retention of 91% (Fig. 2d). These results contrast with the poorer capacity retention observed for VTe in the Zn half-cell after 20 cycles (*i.e.*, 61%) (Fig. S7†). Therefore, these data suggest that potential success in the implementation of VTe in AZIBs will most likely require, at least, further optimisation of the Zn anode. Despite the good capacity retention, a large voltage hysteresis was observed between the charge and discharge plateau (Fig. 2c). We attribute this large voltage hysteresis to high energetics/structural re-ordering occurring in the material, and slow kinetics during Zn^2+^ ion (de)insertion.^35^ This may be reduced by optimising the electrolyte solution, tailoring the crystal size, morphology, and carbon coating of the material, among others.^36,37^

**Fig. 3:**
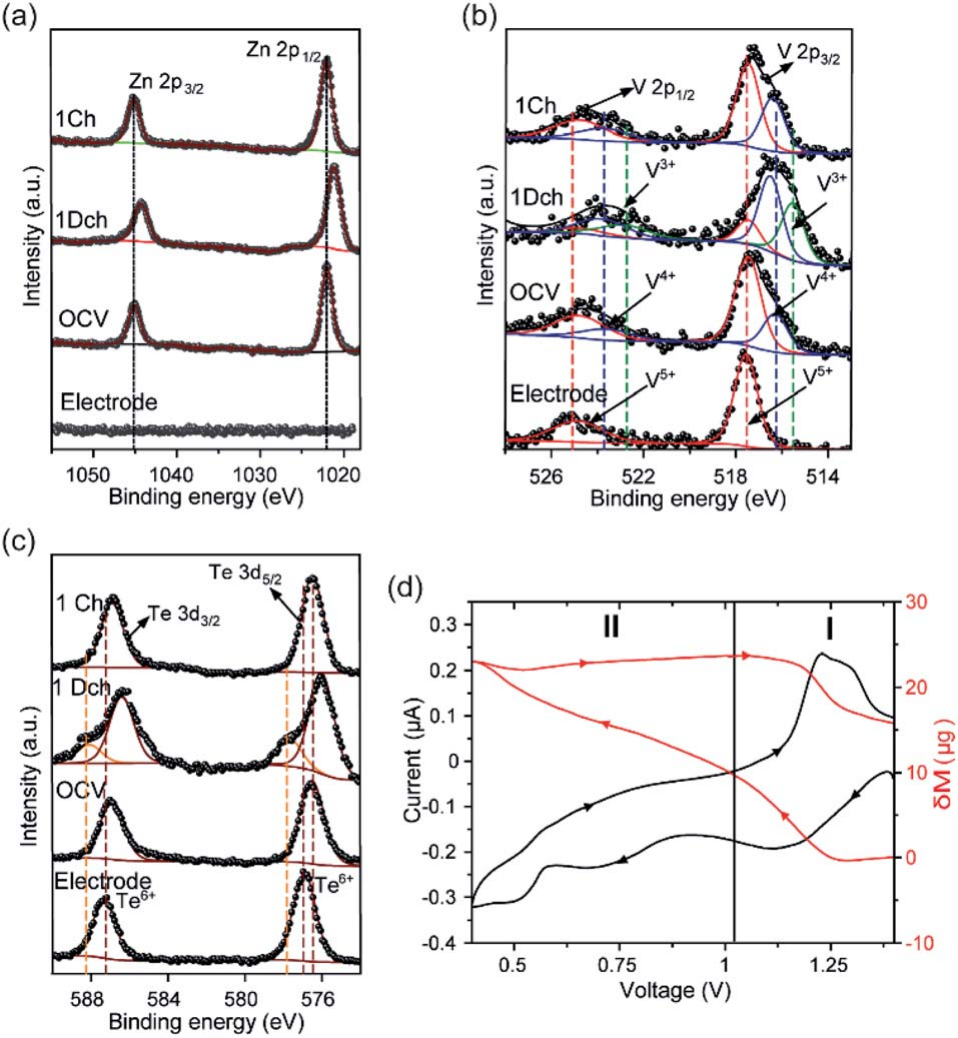
(a) Normalised Zn-2p XPS spectra, (b) V-2p XPS spectra, and (c) Te-3d XPS spectra of VTe at different states of charge. Electrode refers to the pristine electrode and OCV, 1Dch and 1Ch refer to the electrodes extracted from the cell after resting for 6 h, 1^st^ full discharge and charge cycles, respectively. (d) EQCM and corresponding CV data of VTe-coated electrode *vs.* Zn^2+^/Zn at 0.1 mV s^−1^ in 3 M ZnSO_4_ electrolyte solution.

Zn 2p XPS spectrum of the electrode after 6 h under rest conditions (OCV) confirmed the presence of Zn^2+^ ions by showing two asymmetric peaks with binding energies of 1021.5 and 1044.6 eV, assigned to Zn 2p_3/2_ and Zn 2p_1/2_ core levels, respectively (Fig. 3a). Furthermore, the N1s XPS spectrum of the OCV sample showed a decrease in the intensity of the N1s peak (centred at 401.77 eV in the pristine electrode and indicative of the presence of NH_4_^+^ ions), which confirmed that almost all the NH_4_^+^ ions acting as pillars between the {[VO_2_]_2_[Te_2_O_8_(OH)_2_]}^4−^ chains are removed at this stage (Fig. S11†). Nevertheless, it needs to be noted that XPS is a surface analysis technique and that some NH_4_^+^ ions could have been trapped in the bulk structure. Therefore, these data demonstrate that VTe is not completely stable in contact with the 3 M ZnSO_4_ aqueous mild electrolyte solution, and that some pre-intercalation of Zn^2+^ ions takes place before applying current. Charge neutrality is then achieved by the reduction of V^5+^ ions to V^4+^, as observed in the V-2p XPS spectrum (Fig. 3b), where two peaks with maxima at 517.4 and 516.3 eV that correspond to the coexistence of V^5+^ and V^4+^ oxidation states were observed.

By contrast, the V-2p XPS spectrum of the pristine electrode showed a single V 2p_3/2_ peak with a binding energy of 517.5 eV and a broader V 2p_1/2_ spin–orbit pair at higher binding energy, *i.e.*, 524.9 eV, which are typical for V^5+^ ions in oxides.^6,38,39^

Preliminary operando EQCM studies were conducted to identify active charge carriers during Zn^2+^ ion intercalation (Fig. 3d, S12 and Table S2†). During the initial stages of discharge (Region I), the mass change per charge transfer (Δ*m*/Δ*C*) matched with the theoretical value of *ca.* 1.1 Zn^2+^ (347.89 μg C^−1^), implying that Zn^2+^ ions were inserted in their non-hydrated form. These data agrees with the cathodic peak observed in the CV data for the reduction of V^5+^ ions to V^4+^ (Fig. 2a). Region 2, which coincides with the second cathodic peak related to the further reduction of V^4+^ ions to V^3+^, corresponds to a total charge value of 309 μg C^−1^, equivalent to the insertion of 0.9 Zn^2+^ ions. During charge, the reverse process occurs, showing extraction of only one Zn^2+^ based on the Δ*m*/Δ*C* value measured (292 μg C^−1^), in agreement with the Zn 2p XPS spectrum, which shows that there are remaining Zn^2+^ ions upon charge (Fig. 3a) and that all V^3+^ ions have been oxidised to V^4+^and V^5+^ ions (Fig. 3b).

N1s XPS spectra of the fully discharged and charged electrodes showed that the removal of NH_4_^+^ ions was irreversible upon Zn^2+^ intercalation (Fig. S11†). Similar behaviour was reported in (NH_4_)_2_V_6_O_16_·0.9H_2_O, where it was noted that Zn^2+^ insertion irreversibly displaced the NH_4_^+^ ions from the structure.^40^ FTIR data agreed with the XPS data, showing a broad peak at about 3153 cm^−1^ corresponding to the N–H asymmetric stretching mode of the NH_4_^+^ ions, which is no longer present in the fully discharged and charged states (Fig. S13†).^40^ Upon discharge to 0.4 V, the Zn 2p peaks present in the Zn-2p XPS spectrum of the OCV sample shifted to lower binding energies (Fig. 3a). This is explained with a more covalent environment than that in the OCV state. Concomitant to the insertion of Zn^2+^ ions into VTe, we observed a reduction of the V^5+^/V^4+^ redox couple to V^5+^/V^4+^/V^3+^, which explains the broadening and shift toward lower binding energies of the V 2p_3/2_ and V 2p_1/2_ peaks in the V-2p XPS spectrum (Fig. 3b).^6,38,39^ Furthermore, EDX data showed a qualitative increase in Zn^2+^ ions in the discharged state compared to the charged state (Table S3†). The extra discharge capacity observed in the galvanostatic data might be accounted for the irreversible formation of insulating by-products such as zinc hydroxide sulfate (ZHS) salts at the electrode surface due to electrolyte decomposition.^41,42^ IR spectrum of the discharged sample showed strong peaks at 1055 and 1116 cm^−1^, typically attributed to the S–O stretching bands of SO_4_^2−^ moieties in the sample (Fig. S13†).^43^

After charging to 1.4 V, we observed that the Zn 2p_3/2_ and Zn 2p_1/2_ peaks did not fully disappear in the XPS spectrum due to incomplete removal of Zn^2+^ ions (Fig. 3a), confirming the low first coulombic efficiency observed in the galvanostatic data (Fig. 2b). The Zn 2p peaks showed lower binding energy to those observed in the discharged state, which suggests weaker interactions between Zn^2+^ ions and the electrode host.^6^ Zn^2+^ ion extraction was followed by re-oxidation of the majority of the V^4+^ and V^3+^ ions to V^5+^ (Fig. 2b), further confirming the reversible reactions involving the V^5+^/V^4+^ and V^4+^/V^3+^ redox couples observed in the CV data shown in Fig. 2a.

Te-3d XPS spectra of VTe at the pristine state showed two peaks at 576.9 and 587 eV corresponding to the Te-3d_5/2_ and Te 3d_3/2_ core levels, respectively, and characteristic of the binding energy of Te^6+^ ions (Fig. 3c).^44–46^ At the OCV state, we observed a shift of the two peaks to lower binding energy, which we attribute to the presence of different Te environments in the electrode in contact with the electrolyte. For instance, Te^6+^ ions in Te(OH)_6_ and TeO_3_ have binding energies of 576.7 and 577.3 eV.^45,47^ Upon full discharge, these peaks further shifted to lower binding energies, *i.e.*, 576 and 586.4 eV and two additional peaks with maxima at 577.7 and 588.2 eV appeared, which might be attributed to a change in the valence state of Te^6+^ to Te^4+^.^48^ Nevertheless, given the different forms in which Te^6+^ can be present, detailed research is required to unambiguously assigned those peaks to Te^4+^. On the other hand, at the fully charged state, the peaks associated with the Te-3d_5/2_ and Te3d_3/2_ showed almost identical binding energies to those observed at the OCV state, suggesting a reversible process.

*Ex situ* XRD studies were performed on VTe electrodes at different charge states to understand the structural changes taking place during the first cycle (Fig. S14†). XRD data, showed structural changes occurring in the material during the first discharge process, guided by the presence of new main diffraction peaks at 11.9°, 32.3° and 35.6° 2*θ* (marked with a star symbol). Attempts to model the structure of the fully reduced phase were carried out using Expo2014 software.^49,50^ The best possible solution found yielded a phase with a monoclinic crystal structure and lattice parameters *a* = 9.67 Å, *b* = 7.44 Å and *c* = 4.66 Å; α, *γ* = 90° and *β* = 92.3°. No traces of crystalline impurities were observed in the XRD data despite the presence of IR peaks originating from SO_4_^2−^ ions. Therefore, it is likely that these sulfate-containing compounds are amorphous (Fig. S13†).

XRD data of the charged electrode showed a clear loss of crystallinity in the material, as reflected by the peak broadening observed in the diffraction pattern. Nevertheless, some of the diffraction peaks observed in the discharged product were still present, showing that the structure is kept after charge. A detailed diffraction study of the effects of Zn^2+^ insertion/extraction in this material is still required. Based on the above-mentioned experimental results, we propose that the following reactions occur during OCV (eqn (4)), and first discharge (eqn (5)) and charge processes (eqn (6)):

Cathode at the OCV state:

4 (NH_4_)_4_{(VO_2_)_2_[Te_2_O_8_(OH)_2_]}·2H_2_O + *x*Zn^2+^ → (NH_4_)_4−*z*_Zn_*x*_{(VO_2_)_2_[Te_2_O_8_(OH)_2_]}·*n*H_2_O + *z*NH_4_^+^

Cathode during the first discharge:

5 (NH_4_)_4−*z*_Zn_*x*_{(VO_2_)_2_[Te_2_O_8_(OH)_2_]}·*n*H_2_O + (4 − *x* + *y*)Zn^2+^ → Zn_4_(VO_2_)_2_Te_2_O_8+*z*_(OH)_2−*z*_·*n*H_2_O + (4 − *z*)NH_4_^+^

Cathode during the first charge:

6 Zn_4_(VO_2_)_2_Te_2_O_8+*z*_(OH)_2−*z*_·*n*H_2_O → Zn_4−*x*_(VO_2_)_2_Te_2_O_8_(OH)_2_·*n*H_2_O

## 4. Conclusions

In conclusion, we tested (NH_4_)_4_{(VO_2_)_2_[Te_2_O_8_(OH)_2_]}·2H_2_O as a cathode material in AZIBs. Reversible Zn^2+^ ion intercalation in the material is attainable due to the presence of redox centres and large spacing between the 1D {[VO_2_]_2_[Te_2_O_8_(OH)_2_]}^4−^ chains. To the best of our knowledge, this is the first-ever reported vanadium tellurate(vi) tested as a positive electrode in AZIBs. The material shows a remarkable discharge capacity of *ca.* 283 mA h g^−1^ in the 0.4–1.4 V *vs.* Zn^2+^/Zn voltage range, using a current density of 10 mA g^−1^. Furthermore, in concentration cells, VTe shows excellent cycling stability, retaining about 91% of its initial discharge capacity after 20 cycles. Despite the promising results, improvements to the present work are under progress and include various strategies, such as the optimisation of suitable electrolyte formulations, where the V^5+^ state remains stable and prevents the pre-insertion of Zn^2+^ ions, and cycling with a modified Zn anode in a half-cell. Nevertheless, we believe that these results provide great opportunities for the exploration of this material and family-related compounds as energy storage materials for AZIBs.

## Conflicts of interest

There are no conflicts to declare.

## Supplementary Material

RA-012-D2RA01166B-s001
