# Supplementary material for: Synthesis, characterisation, and feasibility studies on the use of vanadium tellurate(vi) as a cathode material for aqueous rechargeable Zn-ion batteries
Source: RSC Adv. 2022 Apr 22;12(19):12211–8. doi: 10.1039/d2ra01166b (PMC9026146; doi:10.1039/d2ra01166b)
Supplement: RA-012-D2RA01166B-s001 [file RA-012-D2RA01166B-s001.pdf]

Electronic Supplementary Material (ESI) for RSC Advances.
This journal is © The Royal Society of Chemistry 2022

Supplementary Information

Synthesis, characterisation, and feasibility studies on the use of vanadium tellurate (VI) as a cathode material for aqueous rechargeable Zn-ion batteries

Mangayarkarasi Nagarathinam[a], Cindy Soares[a], Yue Chen[b], Valerie R. Seymour[a], Vlastimil Mazanek[c], Mark A. Issacs[d,e], Zdenek Sofer[c], Oleg Kolosov[b], John M. Griffin[a], and Nuria Tapia-Ruiz[a, †]

[a] Department of Chemistry, Lancaster University, LA1 4YB, UK.
[b] Department of Physics, Lancaster University, LA1 4YB, UK.
[c] Department of Inorganic Chemistry, University of Chemistry and Technology Prague Technicka 5, 166 28 Prague 6, Czech Republic.
[d] Department of Chemistry, University College London, 20 Gordon St, Bloomsbury, London WC1H 0AJ, UK
[e] Harwell XPS, Research Complex at Harwell, Rutherford Appleton Laboratory, Didcot OX11 0DE, UK

†Corresponding author

Table of Contents

Fig. S1..........2

Fig. S2..........3

Fig. S3..........4

Fig. S4..........5

Fig. S5..........6

Fig. S6..........7

Fig. S7..........8

Fig. S8..........9

Fig. S9..........10

Fig. S10..........11

Fig. S11..........12

Fig. S12..........13

Fig. S13..........14

Fig. S14..........15

Table S1..........16

Table S2...........17

Table S3..........18

References..........19

List of Figures

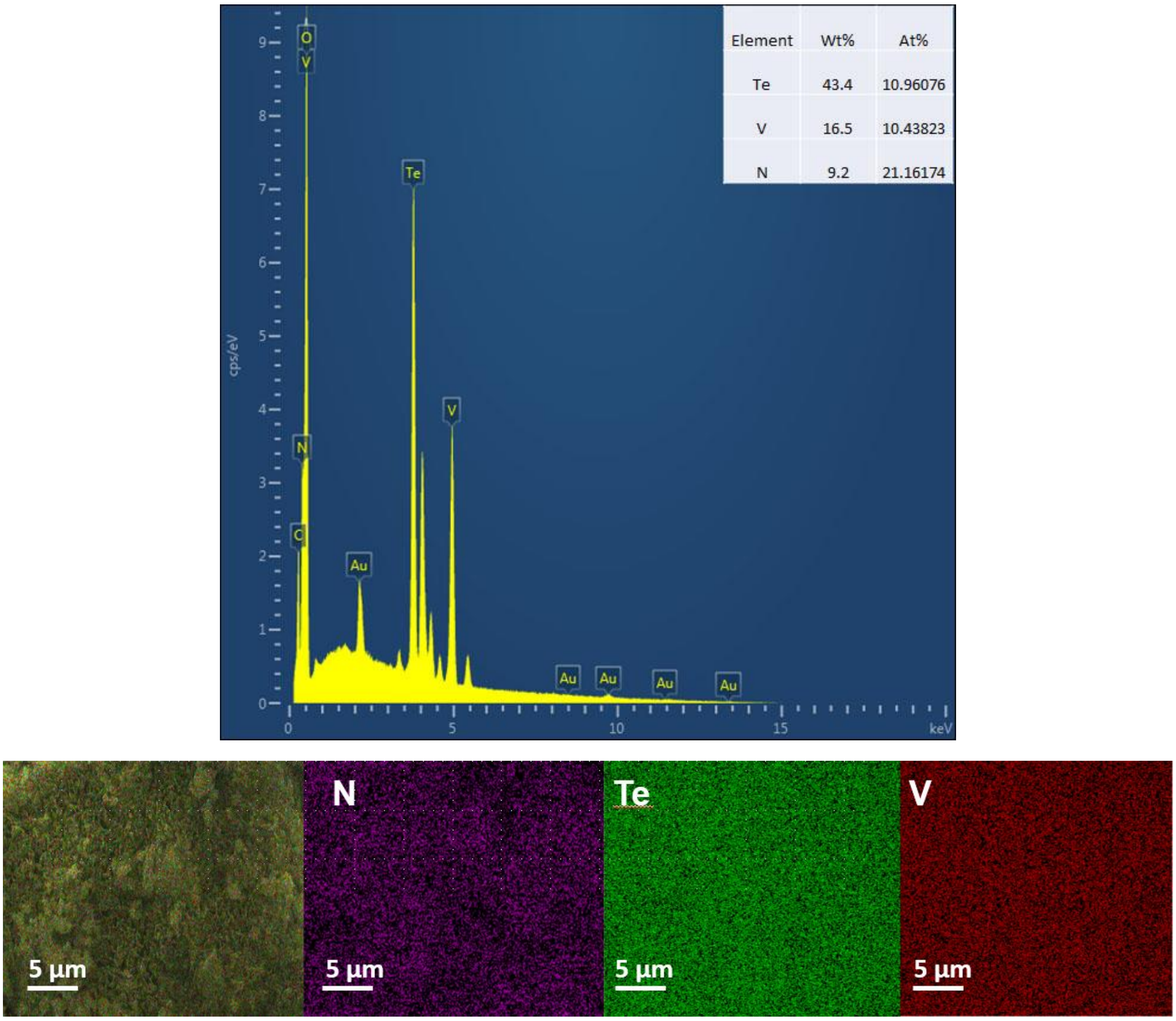

Fig. S1. (Top) EDX spectra of pristine VTe. Au corresponds to sample coating. Inset: Table with wt.% and at% for Te, V and N. (Bottom) EDX image and corresponding EDX mappings showing N, Te, and V atoms.

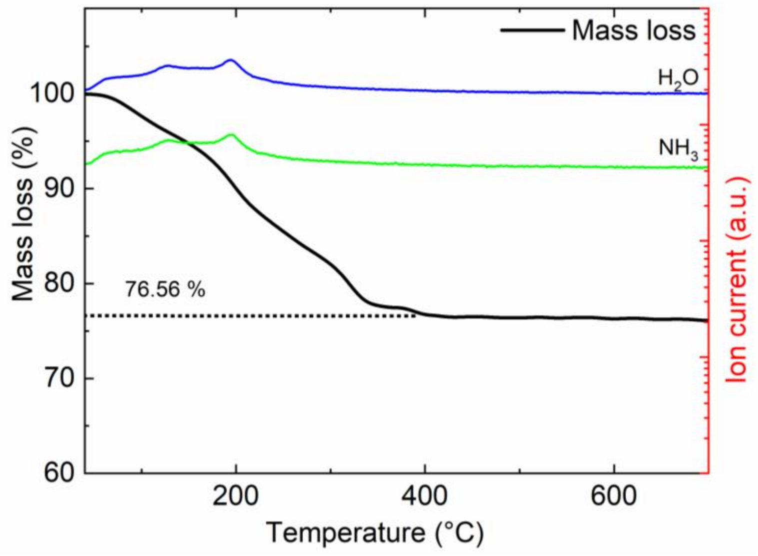

Fig. S2. Thermogravimetric analysis coupled with mass spectrometry data on VTe in the R.T.-700 °C temperature range under an argon atmosphere. The difference between the calculated mass loss related to water and ammonia evolution and the experimental value obtained (*ca.* 6%) might be attributed to extra water molecules in the final product.

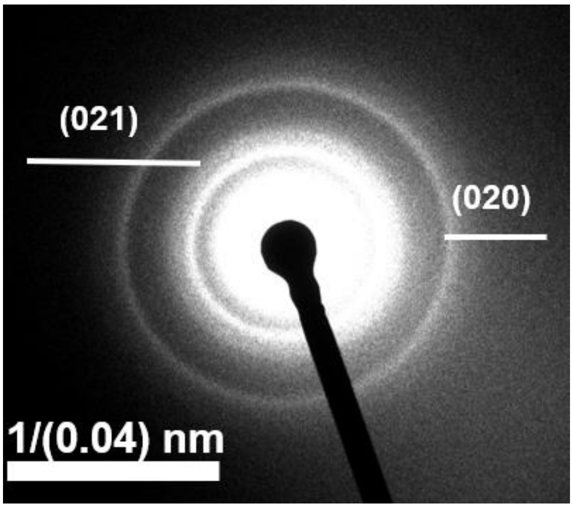

Fig. S3. SAED image of as-synthesised VTe.

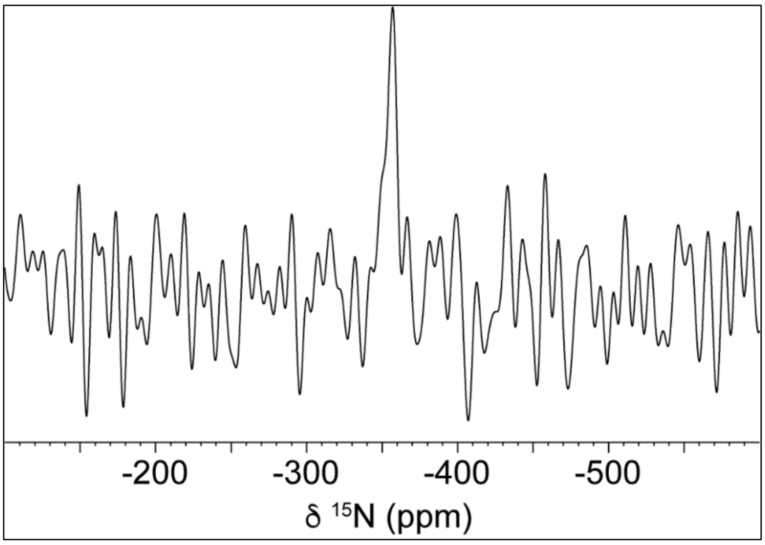

Fig. S4. Experimental ^{15}N MAS NMR spectrum for pristine VTe. The spectrum was acquired with cross-polarisation from ^{1}H.

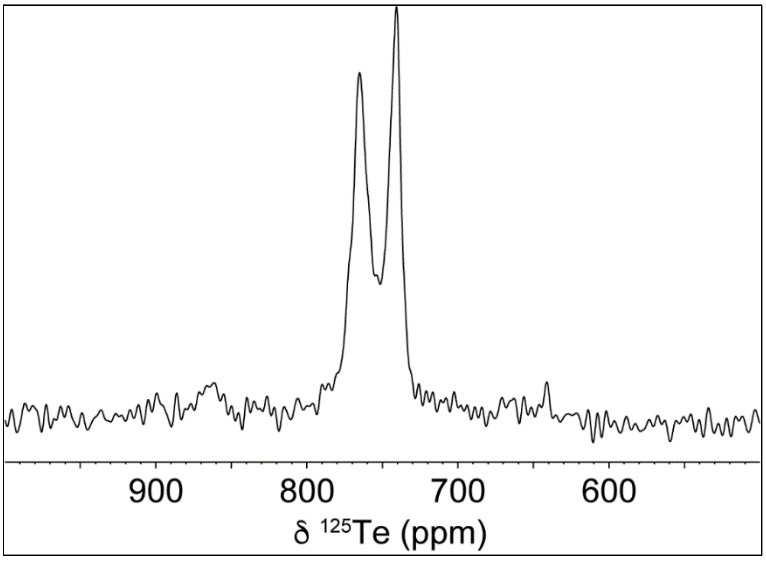

Fig. S5. Experimental ^{125}Te MAS NMR spectrum for pristine VTe.

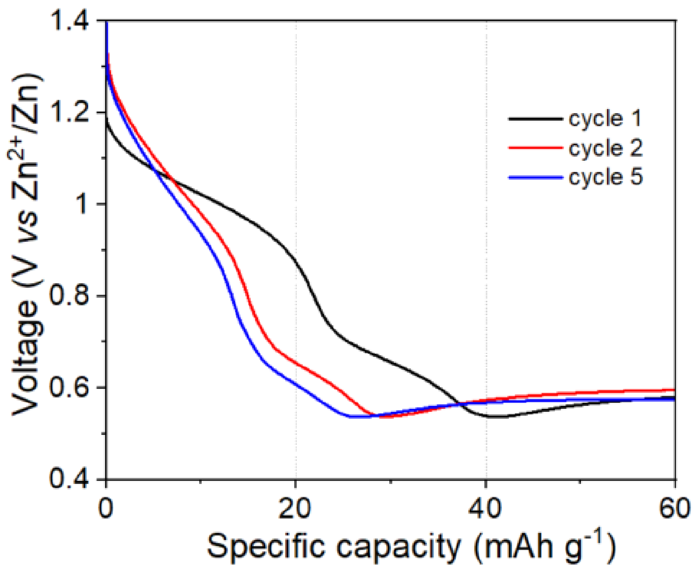

Fig. S6. Magnified view of Fig. 2b, showing galvanostatic discharge profiles of VTe in the 0 to 60 mAh g^{-1} capacity and 0.4-1.4 V vs. Zn^{2+}/Zn voltage ranges, using a current density of 10 mA g^{-1}.

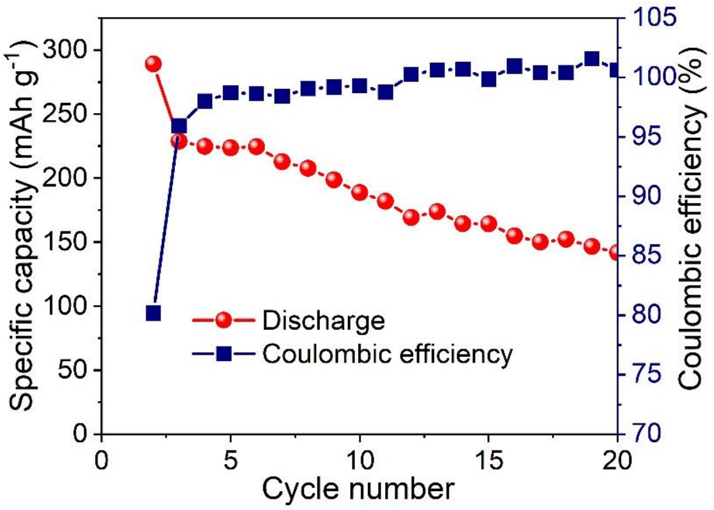

Fig. S7. Specific capacity *vs.* cycle number plot with Coulombic efficiencies of VTe in the 0.4–1.4 V *vs.* Zn^{2+}/Zn voltage range, using a current density of 10 mA g^{-1}.

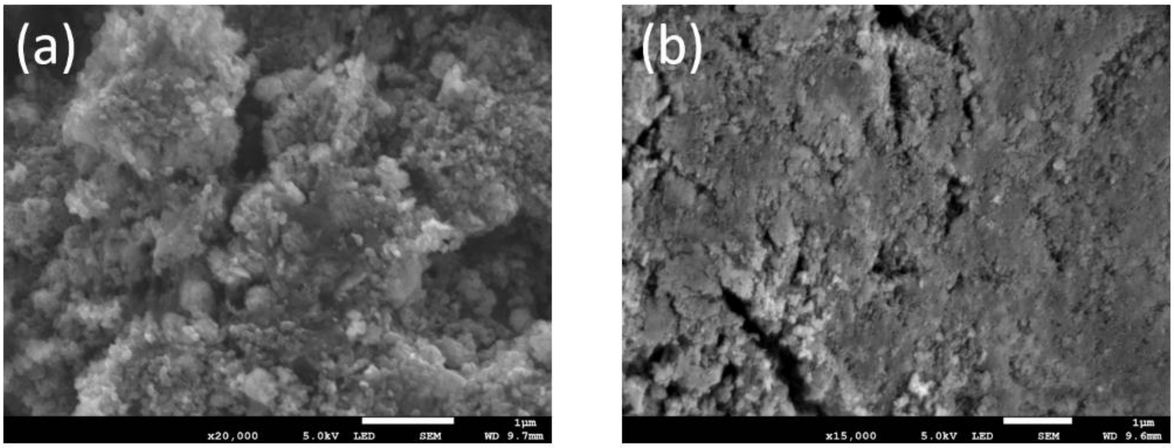

Fig. S8. FESEM images of (a) VTe at OCV (Open Circuit Voltage) state, and (b) after 20 cycles.

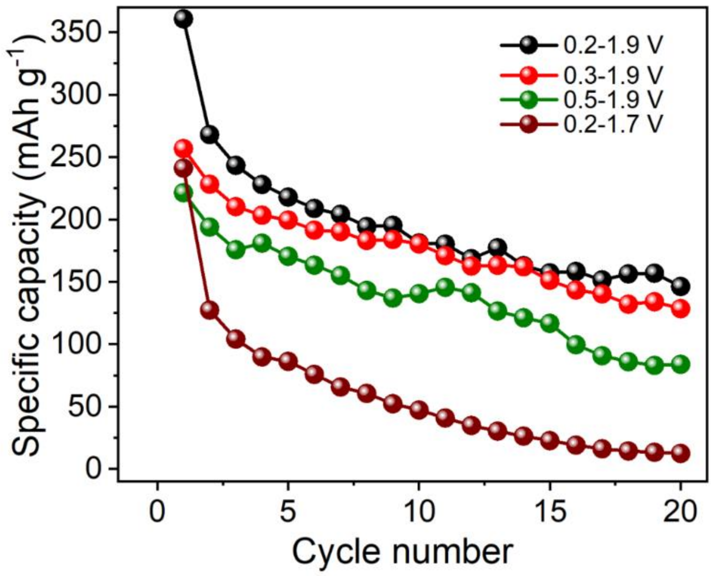

Fig. S9. Specific capacity *vs.* cycle number plots of VTe using different voltage range regimes and a current density of 10 mA g^{-1}.

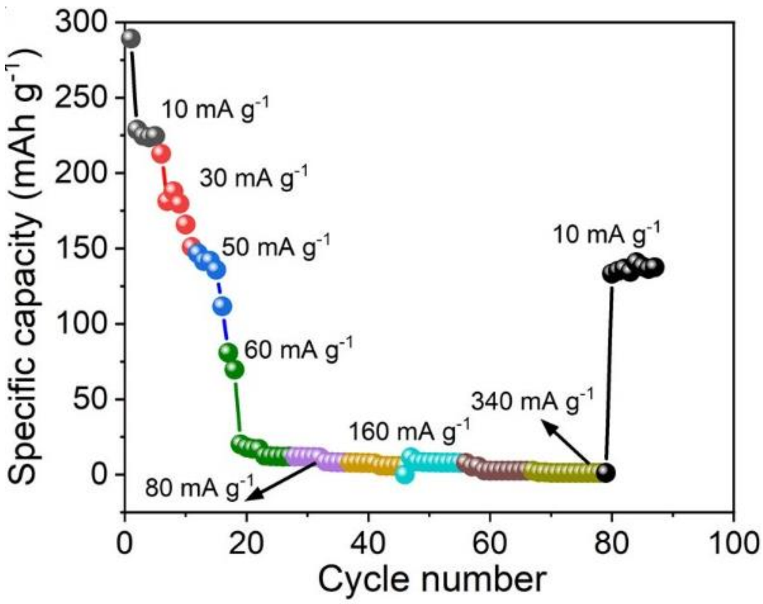

Fig. S10. Rate capability tests of VTe at current densities from 10 to 340 mA g^{-1} in the 0.4–1.4 V *vs*. Zn^{2+}/Zn voltage range.

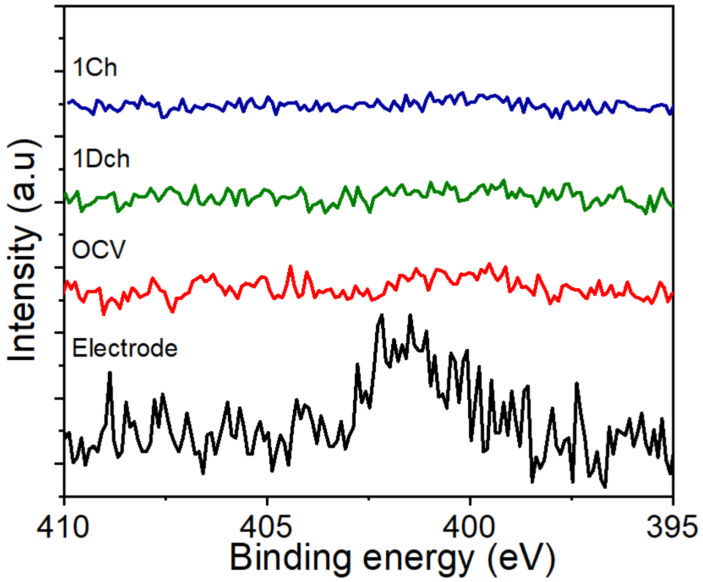

Fig. S11. N1s XPS peak spectra of VTe at different states of charge. Electrode refers to the pristine electrode, and OCV, 1DCh and 1Ch refer to the electrodes extracted from the cell after resting for 6 h, 1st full discharge and charge cycles, respectively.

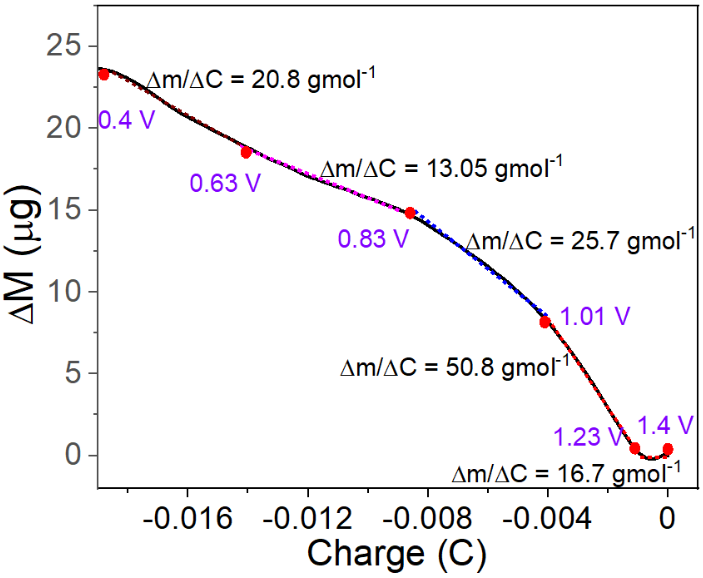

Fig. S12. Mass *vs.* charge curve in the 1.4–0.4 V voltage range. (ΔM/ΔC) slopes were converted into g mol^{-1} for convenience.

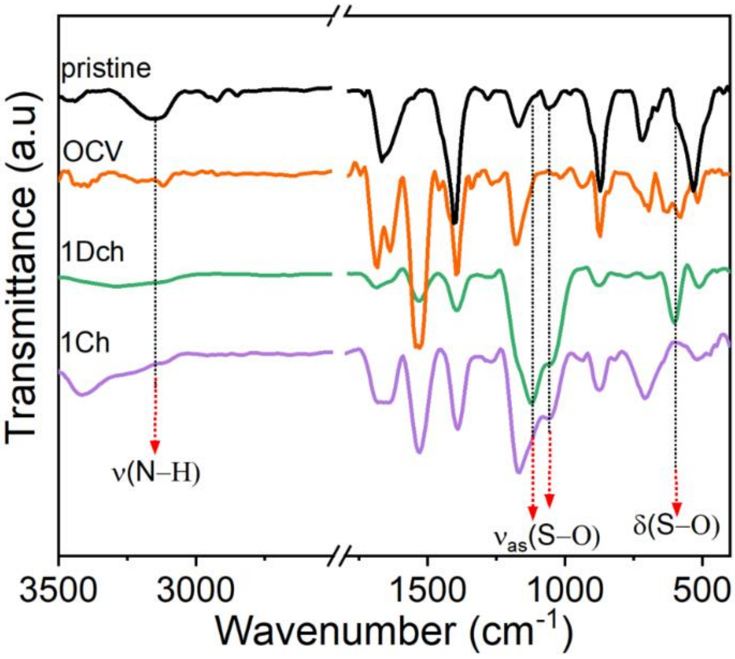

Fig. S13. FTIR spectra of VTe pristine electrode, VTe electrode at the OCV state (after resting for 6 h) and VTe cycled electrodes after full discharge (1Dch) and charge (1Ch). It was not possible to make any assumptions on the presence of NH_4^+ ions at different charged states based on the absence/presence of the N-H symmetric bending vibration mode at ca. 1402 cm^{-1} since this band overlaps with other bands corresponding to stretching and bending vibrations of OH groups.[1]

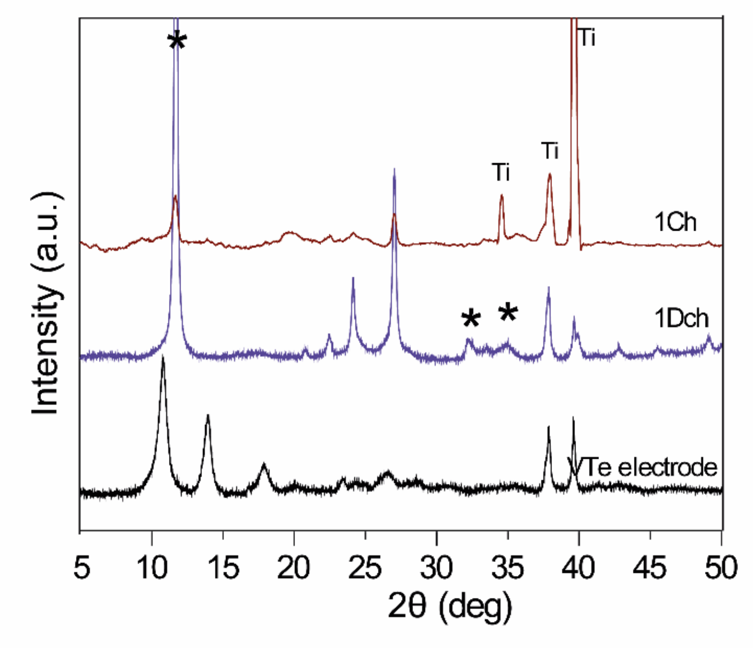

Fig. S14. Ex-situ XRD data of VTe in the pristine, full discharged and charged states.

List of Tables

Table S1. Calculated ^1H chemical shifts for VTe based on the 416841 ICSD crystal structure.[2]

Site	Calculated ^1H chemical shift (ppm)	Experimental ^1H chemical shift (ppm) ± 0.2 ppm
H_2O	4.8	5
NH_4^+	7.2	7
OH	10.1	10

Table S2. Calculated molar mass and equivalent moles of Zn obtained from operando EQCM on the VTe electrode.

CV	$\Delta m/\Delta C$ (μg C^{-1})	Exp. Mw of Zn (g mol^{-1})	Calc Mw of Zn (g mol^{-1})	Calc. Zn mols
**Dch-Region I**	86.69	16.73	16.62	0.26 Zn
**Dch-Region I**	261.20	50.41	51.14	0.8 Zn
**Dch-Region-II**	133.30	25.73	25.57	0.4 Zn
**Dch-Region-II**	67.65	13.06	13.42	0.21 Zn
**Dch-Region-II**	107.90	20.83	18.87	0.32 Zn
**Ch-Region-II**	41.87	8.08	7.86	0.12 Zn
**Ch-Region-II**	37.56	7.25	7.03	0.1 Zn
**Ch-Region-II**	26.06	5.03	6.3	0.1 Zn
**Ch-Region I**	187.3	36.15	38.4	0.6 Zn

Table S3. EDX data of VTe pristine and cycled electrodes after full discharge and charge. Please note that these data may only be used for qualitative purposes.

Samples	Te Atomic %	Zn Atomic %
**Electrode**	4.89	0
**1Dch**	4.15	11.03
**1Ch**	4.17	6.45

References

1 N. V. Chukanov, R. K. Rastsvetaeva, S. M. Aksenov, I. V. Pekov, D. I. Belakovskiy, G. Blass and G. Möhn, *Geol. Ore Depos.*, 2013, **55**, 663–668.K.

2 Hyejin, C. Yoonsuk, Y. Hoseop and D. Junghwan, *Zeitschrift fur Anorg. und Allg. Chemie*, 2007, **633**, 473–477.
